# Demethoxycurcumin analogue DMC-BH exhibits potent anticancer effects on orthotopic glioblastomas

**DOI:** 10.18632/aging.103981

**Published:** 2020-11-18

**Authors:** Lei Shi, Guan Sun, Yong Zhang

**Affiliations:** 1 Department of Neurosurgery, Affiliated Kunshan Hospital of Jiangsu University, Suzhou 215300, P.R. China; 2 Department of Neurosurgery, The Fourth Affiliated Hospital of Nantong University, Yancheng City First People's Hospital, Yancheng 224000, P. R. China; 3 Department of Neurosurgery, Jiangsu Province Academy of Traditional Chinese Medicine, Nanjing 210029, P.R. China

**Keywords:** DMC-BH, demethoxycurcumin, glioblastoma, proliferation, apoptosis

## Abstract

Demethoxycurcumin (DMC) has anti-glioma effects *in vitro* and in subcutaneous xenotransplanted tumors. Our previous study confirmed that the molecule also has mild anti-glioma effects on orthotopic glioblastomas *in vivo*. In this study, we found that DMC-BH, a DMC analogue, exhibited more potent *in vitro* and *in vivo* activities than did DMC. DMC-BH was cytotoxic against various glioma cells including SHG-44, C6, U251, U87, A172 and primary glioma cells. DMC-BH activity was characterized by low acute toxicity and an appropriate pharmacokinetic profile. We evaluated the anti-tumor effects of DMC-BH in an ectopic xenograft model, an orthotopic glioblastoma xenograft model and a patient-derived tumor xenograft (PDTX) model. DMC-BH exhibited potent anti-tumor activity in both the ectopic xenograft and PDTX models. Indeed, bioluminescence measurements showed that DMC-BH exerted a significantly greater anti-tumor effect on orthotopic glioma growth than DMC. Immunohistochemical analysis revealed that DMC-BH inhibited expression of Ki67 and increased the incidence of TUNEL-positive cells. Western blotting showed that DMC-BH significantly decreased p-Akt and p-mTOR expression in orthotopic glioma tissues. These results suggest that the DMC analogue DMC-BH has potent anti-tumor properties that warrant further study.

## INTRODUCTION

Tumors derived from neuroepithelial tissue are collectively referred to as gliomas and account for between 40% and 50% of brain tumors [1]. Malignant glioma is one of the most common intracranial primary tumors, and it is the second most common malignant tumor in children [2]. In the past 30 years, the incidence of primary malignant brain tumors has been increasing, with an annual growth rate of about 1.2%, particularly in the middle-aged and elderly population [3, 4]. Recurrence of glioma is common. Conventional treatment includes surgery, radiotherapy and chemotherapy. However, the 5-year survival rate, especially in patients with glioblastoma, is still very low. A prospective database study of glioblastoma patients treated between May 2007 and December 2014 found that the median survival time was 17.0 months (95% CI: 15.4-18.6) [5].

Curcumin is a phenolic pigment extracted from the rhizome of the *Curcuma longa* plant. It has been shown to be a safe and nontoxic anti-tumor drug [6]. The National Cancer Institute of the United States has listed it as a third-generation chemopreventive agent for use in cancer treatment. Curcumin consists of 3 chemically active monomers, curcumin (C), demethoxycurcumin (DMC) and bisdemethoxycurcumin (BDMC) [6]. Luthra et al. found that DMC is the most potent anti-gliomal component of the three [7]. In our previous research, we discovered that DMC potently inhibits gliomal growth *in vitro* and *in vivo*, and its effects were superior to temozolomide (TMZ) [8, 9]. TMZ is a 3-methyl derivative of mitozolomide, and it is considered to be the first-line anti-glioma chemotherapy drug. Our study also confirmed that DMC could inhibit the proliferation of glioma stem cells [10].

**Figure 1:**
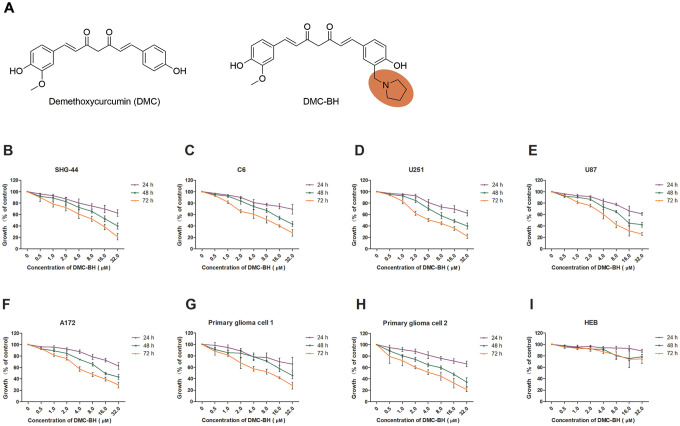
**DMC-BH inhibited the viability of different glioma cells but had minor effects on normal (HEB) cells.** (**A**) The chemical structures of DMC and DMC-BH. (**B**–**H**) An MTT assay showed that DMC-BH inhibited the viabilities of SHG-44, C6, U251, U87, A172 and primary glioma cells in a dose- and time-dependent manner. (**I**) An MTT assay showed that treatment with DMC-BH had little toxic effect on normal (HEB) cells. The values are expressed as mean ± SEM (n = 3 per group).

DMC is characterized by high activity, low toxicity, and relative safety. However, both DMC and curcumin have the disadvantages of poor stability and low bioavailability. In a preliminary experiment, we found that DMC does not effectively pass through the blood-brain barrier (BBB) or exert anti-glioma effects in an orthotopic glioblastoma xenograft model. Therefore, the synthesis of structural DMC derivatives with improved biological activity has become the main direction of our research. Among them, a compound named DMC-BH (Figure 1A) exhibited high chemical stability and significant anti-cancer activity. Thus, we investigated the anti-cancer properties of DMC-BH in glioma cell lines and various mouse models. Studies were also performed to examine the ability of DMC-BH to cross the BBB and to inhibit *in vivo* tumor progression in an orthotopic glioblastoma xenograft model and PDTX model. Using bioluminescent imaging (BLI), we found that the inhibitory effect of DMC-BH on orthotopic glioblastomas is significantly higher than that of DMC, which was confirmed by immunohistochemical studies. In addition, DMC-BH crossed the intact BBB and showed more potent anti-tumor activity than TMZ in a xenograft mouse model using patient-derived glioma-initiating cells.

## RESULTS

### DMC-BH inhibited the viability of glioma cells

To investigate the cytotoxicity of DMC-BH on glioma cells (Figure 1A), we used an MTT assay to assess viability changes in SHG-44, C6, U251, U87, A172 and primary glioma cells treated with various concentrations of DMC-BH for 24, 48 or 72 hours (Figure 1B–1H). DMC-BH dramatically decreased the viability of the SHG-44, C6, U251, U87, A172 and primary glioma cells in a concentration- and incubation time-dependent manner in comparison with control cells. U251 and SHG-44 cells were more sensitive to DMC-BH. We then used the human glial cell line, HEB, to evaluate the toxicity of DMC-BH in normal cells [11]. An MTT assay showed that the viability of HEB cells was not reduced markedly after 24 hours of incubation with 32 μM DMC-BH (Figure 1I). DMC-BH was not toxic to normal cells. At 72 hours, the IC_50_ values of DMC-BH for SHG-44, C6, U251, U87, A172 cells and primary glioma cells (samples 1 and 2) were 3.08 μM, 5.79 μM, 2.52 μM, 4.77 μM, 5.68 μM, 8.05 μM and 4.61 μM, respectively.

### Acute DMC-BH toxicity in mice

To evaluate the safety of DMC-BH, groups of female ICR mice were injected intraperitoneally with a single dose of DMC-BH at 80, 64, 51.2, 40.96, or 32.77 mg/kg or with a vehicle control. The calculated LD_50_ value was 48.0628 mg/kg (43.6668 mg/kg ~ 52.9014 mg/kg), indicating that DMC-BH has a certain degree of acute toxicity on ICR mice. However, further study showed that treatment with DMC-BH at a concentration of less than 20 mg/kg caused no abnormalities compared to the control mice (Table 1).

**Table 1:** DMC-BH toxicity in mice.

**Group**	**Dose (mg/kg)**	**No. of mice**	**No. of dead mice**	**Mortality rate (%)**
1	30	10	1	10.0
2	20	10	0	0.0
3	10	10	0	0.0
4	5	10	0	0.0

### DMC exhibited appropriate pharmacokinetic (PK) profiles

Rat PK studies were performed to study whether DMC-BH is more bioavailable than did DMC. DMC-BH exhibited an approximately 6-fold increased exposure when administered orally compared to DMC [area under the curve (AUC) = 3621 h*ng/mL versus 620 h*ng/mL], suggesting that DMC-BH is more bioavailable (Table 2). The half-life of DMC-BH was longer than that of DMC (7.82 hours versus 4.32 hours). In addition, the maximal concentration (*C*_max_) of DMC-BH was 1264 ng/mL, while the *C*_max_ of DMC was 216 ng/mL. Overall, the bioavailability of DMC-BH was 57.1%, whereas the bioavailability of DMC was 8.3%. DMC-BH exhibited moderate clearance, moderate volume of distribution and acceptable bioavailability. In addition, DMC-BH revealed an excellent plasma protein binding profile (96.2%) and low CYP-450-related metabolism.

**Table 2:** Pharmacokinetic parameters for *in vivo* DMC and DMC-BH.

**Compound No.**	**DMC-BH**		**DMC**	
Route	i.v.	i.g.	i.v.	i.g.
Number of rats	3	3	3	3
Dose (mg/kg)	1.00	10.0	1.00	10.0
T_1/2_ (h)^a^	2.54 ± 0.23	7.82 ± 1.54	1.24 ± 0.21	4.32 ± 1.98
T_max_ (h) ^b^	0.08 ± 0.00	1.45 ± 0.35	0.08 ± 0.00	0.67 ± 0.12
C_max_ (ng/mL)^c^	938 ± 231	1264 ± 385	754 ± 275	216 ± 46
AUC_last_(h*ng/mL)^d^	634 ± 127	3621 ± 623	742 ± 254	620 ± 271
Vz_F_obs (L/kg)^e^	4897 ± 783	54380 ± 19065	5421 ± 432	6543 ± 687
Cl_F_obs^f^ (mL/min/kg)^g^	1475 ± 257	6754 ± 579	1298 ± 220	765 ± 98
MRT_last_ (h)^h^	1.85 ± 0.21	4.21 ± 0.16	1.32 ± 0.29	3.12 ± 0.45
F (%)^i^	57.1%		8.3%	

^a^Half-life. ^b^Maximum plasma concentration time. ^c^Maximum plasma concentration. ^d^Area under the curve. ^e^Volume of distribution. ^f^Plasma clearance rate. ^g^Systemic clearance. ^h^Mean residence Time. ^i^Percent oral bioavailability.

### DMC-BH exhibited appropriate octanol-water partition coefficients and BBB penetrability

**Figure 2:**
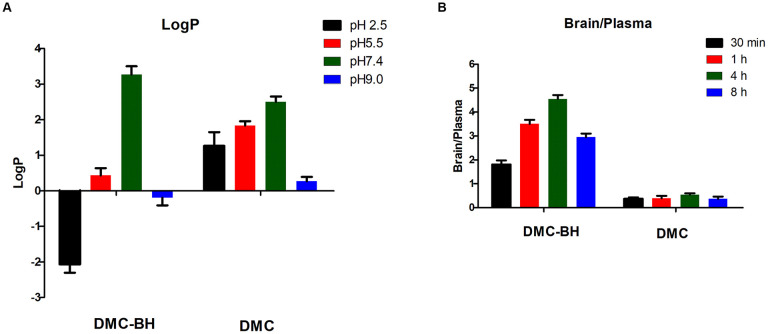
**Octanol-water partition coefficients (logP) and BBB penetrability of DMC-BH.** (**A**) The logP was defined as log(*C*_octanol_/*C*_water_). The amount of the compound in the water phase was determined spectrophotometrically, and the amount in the octanol phase was obtained by subtracting the supernatant concentration from the total concentration. (**B**) The BBB penetrability was defined as a brain-to-plasma AUC_(time point)_ ratio. Values are expressed as the means ± SEM of 3 independent experiments.

To determine whether DMC-BH inhibits the growth of glioma in the brain, we assessed its octanol-water partition coefficients and BBB penetrability. The hydrophobic parameters (logP) of DMC-BH and DMC were estimated under physiological as well as in acidic and basic conditions. The logP values for DMC-BH were negative at pH 2.5 and pH 9.0, as it was an amphoteric compound. At low pH or high pH conditions, DMC-BH occurred in the form of salts, and its logP values became negative due to better solubility. Under physiological conditions (pH 7.4), the logP value of DMC-BH was 3.26, which was appropriate for BBB penetration (Figure 2A). The distribution of DMC-BH to the brain was extensive, as demonstrated by a brain-to-plasma AUC_(4h)_ ratio of 4.6, while the brain-to-plasma AUC_(4h)_ ratio of DMC was only 0.56 (Figure 2B).

### DMC-BH displayed *in vivo* anti-glioma activity in xenograft models

**Figure 3:**
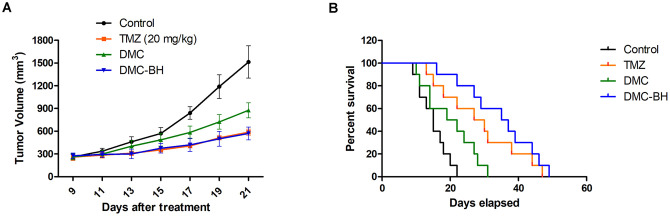
**DMC-BH inhibited U87 glioma cell growth *in vivo*.** Subcutaneous tumors generated from U87 cells were allowed to reach a volume of 150-200 mm^3^ and were treated with 20 mg/kg TMZ, DMC or DMC-BH for 21 days. (**A**) Tumor volume was recorded according to treatment group at the indicated day. (**B**) The survival of brain tumor-bearing mice was recorded and represented in a Kaplan-Meier plot.

To examine the anti-glioma effects of DMC-BH *in vivo*, a human glioma-SCID (severe combined immunodeficient) mouse model was established using the U87 cell line. The mice that received DMC-BH (20 mg/kg) treatment had a lower tumor burden than the control group and a comparable burden to the TMZ (20 mg/kg) group (Figure 3A). The tumor volumes for the control, TMZ, DMC, and DMC-BH mice at day 21 were 1514 mm^3^, 587 mm^3^, 876 mm^3^ and 570 mm^3^, respectively. DMC-BH treatment substantially prolonged the survival of tumor-bearing mice. As shown in the Kaplan-Meier curves, the median survival was 15, 27, 19 and 35 days for the control, TMZ, DMC and DMC-BH groups, respectively (Figure 3B).

### Effects of DMC-BH and DMC on glioblastoma growth in an orthotopic xenograft model

**Figure 4:**
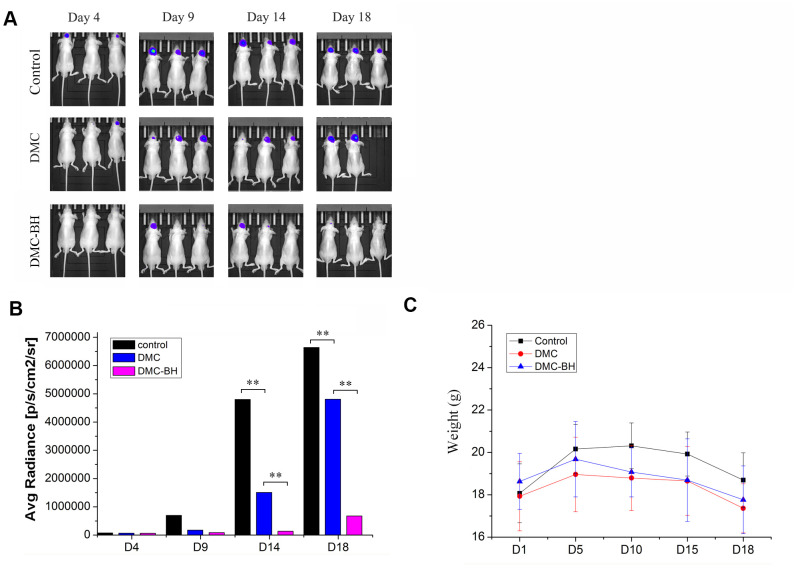
**The effects of DMC-BH and DMC on tumor growth in orthotopic glioblastoma xenograft models.** (**A**) U87-Luc xenograft tumor-bearing nude mice were treated with 20 mg/kg DMC-BH, 20 mg/kg DMC or isometric saline for 18 days. The growth of orthotopic glioblastoma xenografts was monitored by BLI. (**B**) The sizes of orthotopic glioblastoma xenografts were measured using an IVIS spectrum image system. (**C**) The body weight of animals was measured after DMC-BH, DMC or isometric saline treatment.

We used orthotopic glioblastoma xenografts transplanted into mice by human glioblastoma U87-Luc cells to quantify the anti-tumor effect of DMC-BH and DMC. Tumor growth was recorded *in vivo* using Bioluminescent Imaging (BLI). The tumor growth in the control group of saline-treated mice was time-dependently increased (Figure 4A). The tumor-bearing mice treated with 20 mg/kg DMC exhibited slight but significant tumor inhibitory effects 14 days after treatment. However, 20 mg/kg DMC-BH had much greater tumor suppressive effects than the same concentration of DMC. The sizes of the tumors in the mice treated with DMC-BH were much smaller than those in the DMC-treated group or control group (*P* < 0.05) (Figure 4B). These results demonstrated that DMC-BH significantly inhibited intracranial growth of glioblastoma compared with DMC. In addition, no significant body weight loss occurred in any of the three groups (P > 0.05) (Figure 4C).

### DMC-BH inhibits glioma proliferation

**Figure 5:**
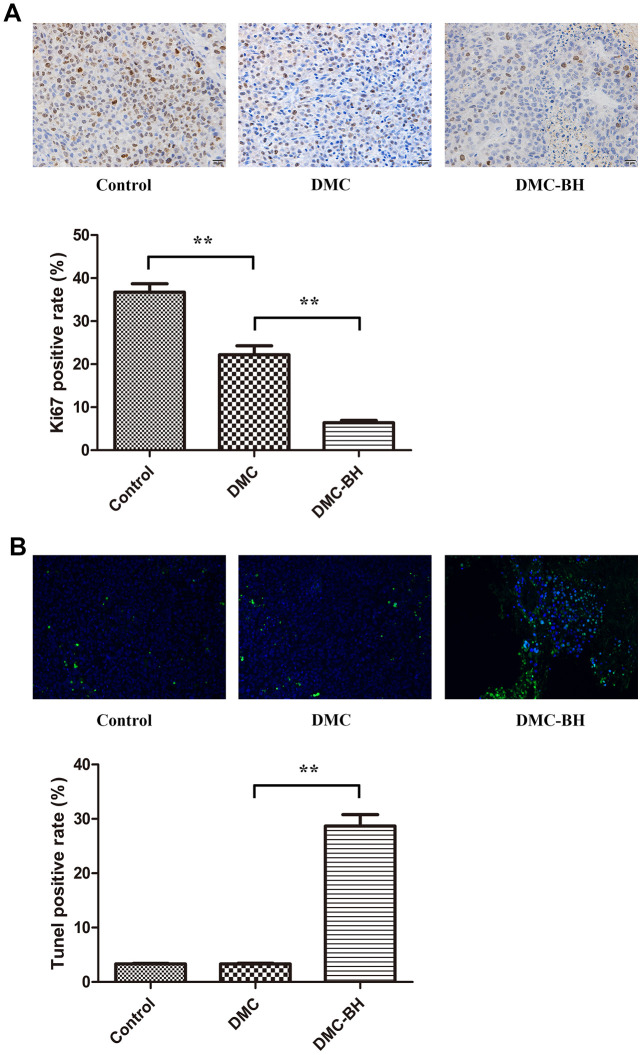
**The effects of DMC-BH and DMC on orthotopic glioblastoma growth were analyzed by immunohistochemical staining.** (**A**) The orthotopic glioblastoma growth potential after treatment with 20 mg/kg DMC-BH or DMC treatment was evaluated by Ki67 immunohistochemistry. (**B**) The orthotopic glioblastoma apoptosis rate after treatment with 20 mg/kg DMC-BH or DMC was analyzed by TUNEL staining.

Tissue samples from the xenograft animals were collected for IHC analysis. The antigen Ki67 was used to evaluate cell proliferation ability. The Ki67-positive rates were 6.38% ± 1.38% in the DMC-BH-treated group and 22.17% ± 5.10% in DMC-treated group (*P* < 0.05) (Figure 5A). The DMC-BH group rate was significantly lower than that of the DMC group (*P* < 0.05). The Ki67-positive rate of in the untreated group was 36.73% ± 4.82%, which was higher than that in DMC-BH or DMC-treated groups. The percentage of TUNEL-positive cells increased in the DMC-BH-treated group (28.68% ± 5.15%) but not in the control (3.30% ± 0.26%) or DMC (3.33% ± 0.31%) groups (*P* < 0.05) (Figure 5B).

### DMC-BH inhibited the Akt/mTOR pathway

**Figure 6:**
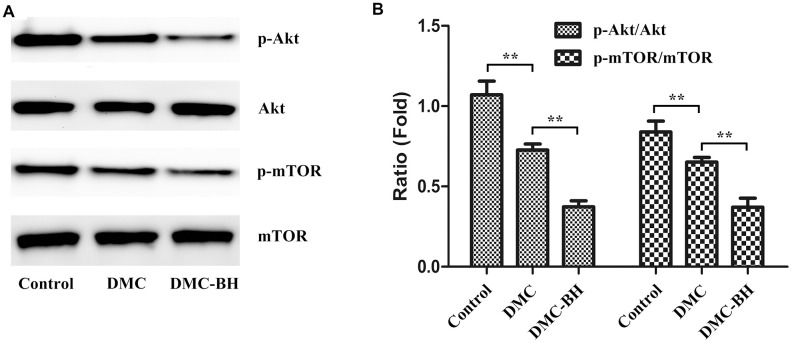
**The effects of DMC-BH and DMC on the Akt/mTOR signaling pathway in U87 orthotopic glioblastoma xenografts.** (**A**) Total protein was extracted from U87 orthotopic glioblastoma xenografts. Then p-Akt and p-mTOR protein expression was detected by Western blot. (**B**) The column chart illustrates the relative expression levels of these proteins.

The PI3K/Akt/mTOR signaling pathway is over activated in glioma tissues, and this increased activation is closely related to malignancy and chemoresistance of gliomas [12, 13]. Previous research reported that among its potential anti-tumor actions in breast cancer, DMC targeted multiple AMPK downstream pathways involving dephosphorylation of Akt and mTOR inhibition [14]. Because DMC-BH is a DMC derivative, we further investigated the inhibitory effects of DMC-BH on gliomas by targeting the Akt/mTOR signaling pathway. We evaluated the expression of p-Akt, and p-mTOR in tumor tissues after DMC-BH treatment (Figure 6A). As shown in Figure 6B, p-Akt and p-mTOR levels were significantly higher after DMC-BH treatment compared with DMC or control treatment (*P* < 0.05). No significant changes were found in total Akt and mTOR expression. Moreover, the expression of p-Akt and p-mTOR in the DMC group was decreased with that in the control group (*P* < 0.05).

### DMC-BH inhibited cell proliferation in a patient-derived tumor xenograft model

**Figure 7:**
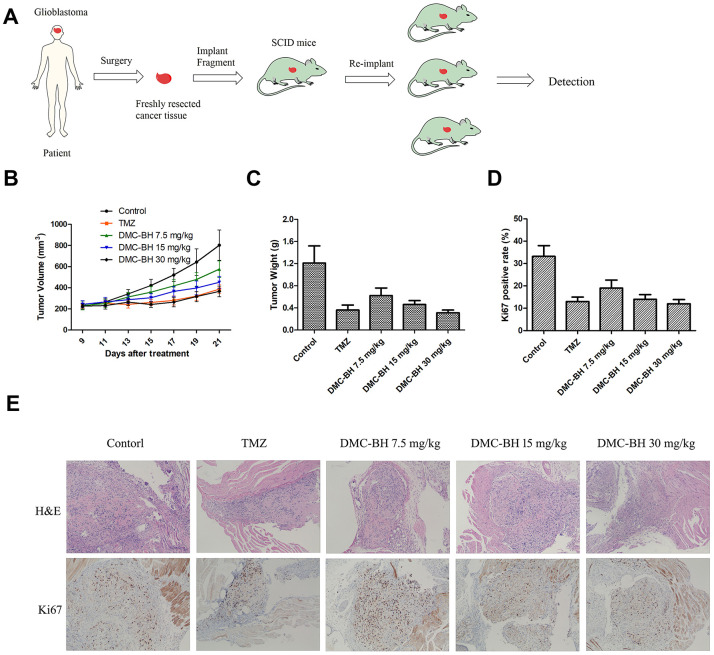
***In vivo* anti-tumor activity of DMC-BH in the PDTX tumor model.** (**A**) A scheme illustrating the process of creating the patient-derived tumor model. (**B**) Tumor growth curves of mice after treatment with 7.5 mg/kg, 15 mg/kg or 30 mg/kg DMC-BH or 20 mg/kg TMZ. (**C**) Average tumor weight at day 21 after different treatments. (**D**) Quantification of the Ki67-positive area in different treatment groups. (**E**) Representative H&E-stained tumor slices and Ki67 immunofluorescence labeled tumor tissue sections from different treatments groups. The data are shown as mean ± SD (n = 6).

In recent years, PDTX models have been used to mimic human tumors. These models are more clinically relevant than cell line-based xenograft models. Thus, we performed an *in vivo* study in a PDTX tumor model by implanting human glioma samples from a patient donor into B-NDG mice (Figure 7A). Treatment of DMC-BH or TMZ was tolerated and caused significant tumor regression in immunocompromised mice bearing glioblastoma multiforme (GBM) patient derived tumors (Figure 7B, 7C). To further demonstrate that DMC-BH can efficiently inhibit the growth of glioblastomas, an ex vivo immunofluorescence staining assay was performed (Figure 7D, 7E). A marked decrease in the number of Ki67-positive cells was observed in the tumor treated with 20 mg/kg DMC-BH compared with the control tumor, suggesting inhibition of tumor cell proliferation likely contributed to the anti-tumor effect of DMC-BH.

## DISCUSSION

Glioblastoma is one of the most common malignant brain tumors and is characterized by fast progression and poor prognosis. Although extensive efforts have been conducted to discover novel drugs and effective molecular targets, the overall treatment on glioblastoma is still very poor. Curcumin is a prospective anti-tumor drug. It has been reported to suppress proliferation of human prostate cancer, breast cancer and lung cancer [15–18]. It was also found to inhibit the proliferation of gliomas [19]. However, its anti-glioma effect was far weaker than that of DMC. Luthra et al. analyzed the anti-glioma activity of curcumin (C1), demethoxycurcumin (C2) and bisdemethoxycurcumin (C3), and revealed that DMC (C2) inhibited glioma cell proliferation and induced apoptosis most effectively [7]. This conclusion was confirmed by Huang et al. [20]. Our previous studies have demonstrated that DMC was superior to TMZ at inhibiting proliferation and inducing apoptosis of glioblastoma cells both *in vitro* and *in vivo* [8–10].

TMZ is one of the first-line chemotherapeutic agents currently recommended for postoperative treatment of gliomas [21]. TMZ can be converted into 5-(3-Methyl-1-triazeno)imidazole-4-carboxamide (MTIC) *in vivo* to penetrate the BBB and exert anti-tumor effects [22]. In our previous research, our data showed that TMZ but not DMC had had a marked anti-tumor effect on orthotopic glioblastomas, indicating that DMC or its *in vivo* metabolite only partially penetrates the BBB [23]. To overcome this shortcoming, we modified the chemical structure of DMC and found a potent derivative of DMC, DMC-BH. In the present study, we investigated the effects of DMC-BH on glioblastomas. DMC-BH had good safety and appropriate PK profiles. *In vivo* anti-tumor experiments showed that DMC-BH had potent anticancer effects against orthotopic glioblastomas, while DMC only modestly inhibited orthotopic glioblastomas. These data indirectly revealed that DMC-BH could easily passes the BBB. The BBB penetration of DMC-BH and DMC was compared by determining the brain-to-plasma AUC ratios. We found that the brain-to-plasma AUC_(4h)_ ratio of DMC-BH was 4.6, while the brain-to-plasma AUC_(4h)_ ratio of DMC was 0.56. The better BBB penetration of DMC-BH and improved anti-tumor activity can be largely attributed to the appended pyrrolidine moiety on the benzene ring of DMC, which results in an appropriate logP value of DMC-BH.

Here we confirmed that DMC-BH was more potent than DMC in inhibiting cell proliferation and inducing apoptosis in orthotopic glioblastoma cells. Further, in immunohistochemical analysis using Ki67 and TUNEL, DMC-BH showed much more potent anti-cancer effects on both cell proliferation and apoptosis than did DMC. No obvious apoptosis was observed in DMC-treated orthotopic glioblastoma tissues. These data suggested that DMC-BH but not DMC effectively passed through the blood-brain barrier to induce cell apoptosis in glioma cells.

In tumor cells, AKT can be transformed into pAKT through phosphatidylinositol 3-kinase phosphorylation, and pAKT can promote cell proliferation and inhibit cell apoptosis by activating caspase 9, Bad, FOXO, GSK-3β and IKK by phosphorylation. Meanwhile, pAKT can also alleviate the inhibition of mTOR by TSC1/2 and Rheb. The activation of mTOR and RAPTOR can activate ribosome S6 kinase through mTORC1 phosphorylation, interfering with the 5 '-TOP structure of mRNA translation and affecting the proliferation of cells [24]. It has been suggested that the Akt/mTOR signaling pathway plays a cytoprotective role in GBM. Zhao et al. showed that cytarabine inhibited leptomeningeal metastasis of high-grade glioma through the PI3K/Akt/mTOR pathway [25]. Liu et al. reported the that hyperactivation of the AKT/mTOR pathway is frequently observed in gliomas, and inhibiting the PI3K/AKT/mTOR pathway could enhance the sensitivity of gliomas to TMZ [26]. In the current study, we confirmed that DMC-BH significantly decreased the expression of p-Akt and p-mTOR compared with DMC and control in orthotopic tumor tissues. These data suggest that the AKT/mTOR pathway plays a role in the anti-glioma effects of DMC-BH.

In summary, this study demonstrates that the novel compound DMC-BH exerts a potent anti-tumor effect on both orthotopic glioblastomas and PDTX models. We demonstrated that the compounds effects appear to involve suppression of the Akt/mTOR signaling pathway.

## MATERIALS AND METHODS

### Cell lines and culturing conditions

The human GBM U87-luc cell line was purchased from the Shanghai Sunbio Medical Biotechnology Co., Ltd. (Shanghai, China). Cells were cultured in DMEM supplemented with 10% fetal bovine serum (FBS) and maintained in an incubator at 37° C and 5% CO_2_. After cells were routinely passaged at 2- to 3-day intervals, animal experiments were carried out when the cells entered exponential growth phase. Rat C6, human SHG-44, U87, A172 and U251 glioma cells were obtained from the Shanghai Institute of Cell Biology, Chinese Academy of Sciences (Shanghai, China). Primary glioma cells were preserved by our laboratory from previous studies. These cells were cultured in DMEM containing 10% fetal bovine serum, 2 mmol/L glutamine, penicillin (100 U/mL) and streptomycin (100 μg/mL). The cells were maintained in an incubator at 37° C and 5% CO_2_. Cells in the mid-log phase were used for experiments.

### Cell viability assay

Glioblastoma cells were seeded into 96-well plates and exposed to vehicle (PBS) or different concentrations of DMC-BH for 24, 48 or 72 hours. Cell viability was measured using an MTT assay and was expressed as a ratio to the absorbance value of the control cells at 570 nm.

### Acute toxicity evaluation

Female Kunming (KM) mice were purchased from Shanghai Experimental Animal Center of Chinese Academy of Science (Shanghai, China) and housed individually in a specific pathogen-free facility. Groups of mice (n = 10 per group) were injected intraperitoneally once with various concentrations of DMC-BH or vehicle control, respectively. Mouse death was monitored and recorded daily up to 14 days after injection. The animal experimental protocols were approved by the Animal Ethics committee of the First People's Hospital of Kunshan.

### Human tumor xenografts in SCID mice

1-[4-Hydroxy-3-(pyrrolidin-1-ylmethyl) phenyl]-7-(4-hydroxy-3-methoxyphenyl) hepta-1,6-diene-3,5-dione, named DMC-BH, was constructed by our lab. DMC was obtained from Sigma-Aldrich (St. Louis, MO). To test the anti-glioma effect of DMC-BH *in vivo*, a xenograft model of human glioma was established. Four-week-old male SCID mice were purchased from Vital River Laboratory Animal Technology Co., Ltd. (Beijing, China). After a 1-week acclimatization, SCID mice were injected subcutaneously in the right flank with 6 × 10^5^ U251 glioma cells resuspended in 50 μL DMEM media. Treatment was initiated when the subcutaneous tumors reached an average size of 150 mm^3^ to 200 mm^3^. Mice received intraperitoneal injection of DMC (20 mg/kg/day), DMC-BH (20 mg/kg/day), TMZ (20 mg/kg/day) or vehicle as a control. Tumor diameter was measured every 2 days with calipers, and the tumor volume was calculated (length × width × height × 0.5). All experiments were carried out in keeping with the procedures and protocols of the Animal Ethics Committee of the First People's Hospital of Kunshan.

### Pharmacokinetic study

Female Sprague-Dawley (SD) rats (n = 3) were purchased from the Shanghai Experimental Animal Center of Chinese Academy of Science (Shanghai, China). After 3 days of acclimatization, DMC-BH or DMC was administered intravenously into the jugular vein catheters at a dose of 1.00 mg/kg. After oral administration, blood samples (250 μL) were collected via jugular vein catheters at 30, 60, 90, 120, 150, 180 and 240 minutes and 8, 12 and 24 hours. Plasma samples were prepared by centrifuging the blood samples at 13,000 g for 5 minutes. All plasma samples were stored immediately at -80° C until analyzed. Analytes were extracted from 100 μL of plasma with 300 μL of acetonitrile containing 125 ng/mL of the internal standard (midazolam). The samples were thoroughly mixed and centrifuged at 8,000 g. The organic extract was added to 150 μL of 5mM NH_4_FA and transferred to the autosampler for LC-MS/MS analysis. Multiple reaction monitoring mode, a highly specific and sensitive label-free technique was performed by scanning the most sensitive signals. The pharmacokinetic parameters were determined using non-compartmental analysis (WinNonlin, Pharsight Corporation, Mountain View, CA, USA).

### Determination of octanol-water partition coefficients

The partition coefficient (P) between *n*-octanol and water was determined. The buffer mixture (0.05 M citric buffer, pH 2.5; 0.05 M acetic acid buffer, pH 5.5; 5 mM PBS, pH 7.4; or 0.05 M bicarbonate buffer, pH 9.0) and *n*-octanol was pre-equilibrated overnight at room temperature. After addition of the DMC or DMC-BH, the mixture was vortexed for 10 minutes and incubated at room temperature for an additional 15 minutes before centrifugation at 1000 g for 5 minutes. The amount of the compound in water phase was determined spectrophotometrically using standard curves, and the amount in lipid phase was obtained by subtracting the supernatant concentration from the total concentration, measured before mixing of the phases. P was determined according to P = (*n_oct_*/*V_oct_*)/(*n_w_*/*V_w_*), where *n* denotes the number of compound moles, *V* is the volume and the subscripts *oct* and *w* refer to *n*-octanol and aqueous phase, respectively.

### Orthotopic glioblastoma xenografts transplanted with U87-luc cells

Thirty-six male nude mice weighing between approximately 18 g and 20 g and aged 6 to 8 weeks were divided into a control group, DMC group and DMC-BH group. The nude mice were handled under a laminar flow hood, and the feed, bedding, cage and contact instruments were all used after high pressure disinfection. The amplified U87-Luc cells were passaged in a 6 cm culture dish and placed in a 5% CO_2_ incubator. Ten percent chloral hydrate (40 μL/10 g) was used to anesthetize nude mice, and the nude mice were fixed on a stereotaxic apparatus when they were under deep anesthesia. We began to digest the U87-Luc cells once the mice were immobilized. The concentration of cells was 1 × 10^8^/mL. Using a micro syringe, we slowly drew up 5 μL cells. Then 5 × 10^5^ U87-Luc cells were injected into the caudate nucleus of the nude mice through an infusion of 1 μL/min. The orthotopic glioblastoma growth was quantified by BLI using an IVIS SPECTRUM 200 system (Perkin Elmer). Either DMC-BH or DMC was administered via intraperitoneal injection at a dose level of 20 mg/kg per injection. The drug administration started 10 days after intracranial implantation of U87-Luc cells, and *in vivo* imaging confirmed that the tumors were formed. The drug was administered continuously for 18 days. Control mice received an equivalent volume of physiologic solution each day. At day 18, all animals were sacrificed by CO_2_ inhalation.

### Bioluminescence measurement

Anesthetize mice with 2-3% isoflurane. Inject mice intraperitoneally with D-luciferin (150 μl of 15 mg/ml stock). Ten minutes after luciferin injection assess bioluminescent signal over an integration time of 10 and 60 s using a cooled charge-coupled device (CCD) camera (Spectrum; Caliper Life Sciences, Hopkinton, MA, USA). BLI was used to monitor tumor growth and response to therapy using the IVIS spectrum imaging system.

### Establishment of a human glioma PDTX tumor model in SCID mice

Surgically resected human glioma tissue was obtained from a 68-year-old cancer patient (#0407002) following an approved Nanjing Medicinal University Institutional Review Board protocol. Within 2 hours of surgical resection, tumor tissues were cut into 1-2 mm fragments and implanted into SCID mice (B-NDG, 6 weeks old, female) using a surgical procedure approved by Nanjing Medicinal University Institutional Animal Care and Use committee. After surgery, tumor growth in the mice was monitored by palpating the abdomen twice per week. Tumors grew to 5-10 mm in diameter in about 8-10 weeks. The SCID mice bearing PDTX tumors were sacrificed, and tumor fragments of 1 to 2 mm in size were implanted into 6-week-old female SCID mice. Over 60% of the mice experienced tumor growth in approximately 6 weeks.

### Immunohistochemistry

Following perfusion, the whole brain was immersed in 10% neutral paraformaldehyde buffer solution and fixed for 48 hours at room temperature. Routine dehydration and paraffin embedding were performed after fixation. Paraffin blocks were sliced to a thickness of 3 μm. We dewaxed tissue sections with xylene and rehydrated them through an ethanol gradient. The sections were then rinsed 2 times for 5 minutes with PBS. Incubate with 3% methanol solution of hydrogen peroxide for 20 minutes. Sections were then incubated in 5% goat serum for 10 minutes at room temperature. Serum was removed, and the sections were incubated with the first antibody at 4° C degree overnight. After PBS flushing, we added a secondary biotin-conjugated antibody. Sections were again flushed with PBS, and streptomycin bacteria antibiotic peroxidase solution was added. Finally, DAB was added and samples were stained with hematoxylin.

### Statistical analysis

All tests were performed using SPSS Graduate Pack 11.0 statistical software (SPSS, Chicago, IL, USA). Descriptive statistics, including the mean and SE, and one-way ANOVAs were used to determine significant differences. *P* < 0.05 and *P* < 0.01 were considered statistically significant.
